# Clear Evidence for Two Rounds of Vertebrate Genome Duplication

**DOI:** 10.1371/journal.pbio.0030344

**Published:** 2005-09-06

As one of the most important sources of novel gene functions, gene duplications play a major role in evolutionary change. Though a gene copy will generally become inactive after duplication, it can be saved—either by acquiring a new function or dividing aspects of the original gene's function—on its way to becoming ubiquitous, or “fixed,” within the population.

The notion of “evolution by gene duplication” was proposed in 1970 by Susumu Ohno, who argued that gene and whole genome duplication provided the raw material for evolutionary innovations such as subcellular compartments, fins, and jaws. Having “extra” copies of genes provides the opportunity for duplicate genes to escape the constraints of purifying selection, and allows the genes to diverge and acquire novel functions. Ohno also proposed that two rounds of whole genome duplication occurred at some point in early vertebrate evolution—a possibility that could explain the relatively large size and complexity of the vertebrate genome.[Fig pbio-0030344-g001]

**Figure pbio-0030344-g001:**
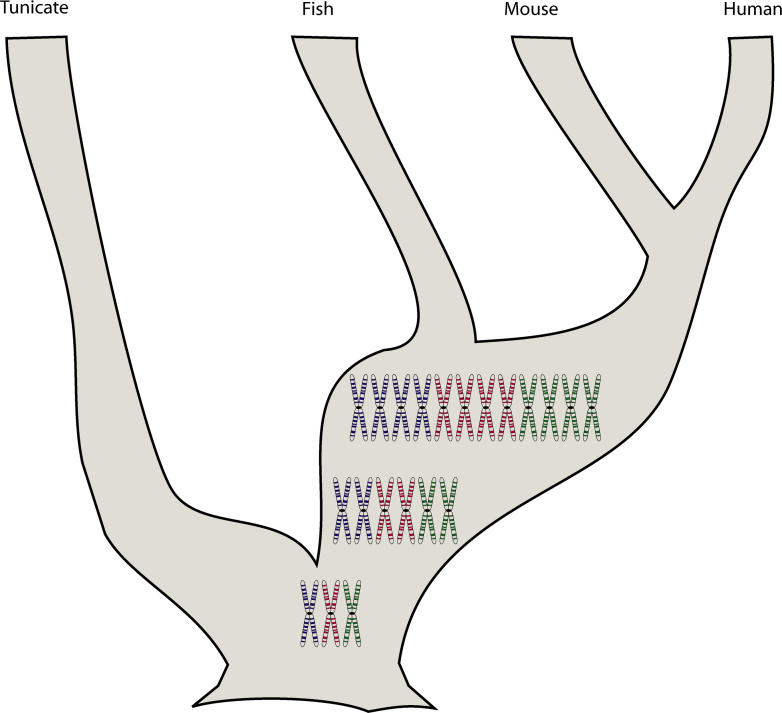
The evolutionary patterns of gene duplications were reconstructed by comparing the complete gene sets of a tunicate (sea squirt), fish, mouse, and human. The 4-fold pattern in their global physical organization provides unmistakable evidence of two distinct, ancient whole genome duplications

Investigators equipped with far more powerful genome-mining tools than were available to Ohno have long sought evidence of this hypothesis (known as the 2R hypothesis, for “two rounds” of whole genome duplication), but with conflicting results. The observation that some gene families have four members in vertebrates but just one in invertebrates (the 4:1 rule) appeared to support the 2R hypothesis, until it was discovered that less than 5% of homologous gene families (similar genes with shared ancestry) followed the rule. But even when gene families do follow the rule, their configuration could just as likely arise from two rounds of single gene duplication as from whole genome duplication. And because duplicate genes are far more likely to degrade than to assume new or shared functions, the signal of whole genome duplication disappears.

Recent studies have shown that the global pattern of the physical location of homologous genes provides evidence of ancient whole genome duplications in yeast and plants, even when most of the duplicates have degraded. Now Paramvir Dehal and Jeffrey Boore have taken this approach to test the 2R hypothesis, by comparing the recently completed genome sequence of the invertebrate sea squirt with the genomes of three vertebrates—pufferfish, mouse, and human. (Because the sea squirt is a close relative of vertebrates, its genome can help reconstruct a more accurate tree of the organisms' evolutionary relationships than a more distant relative like the fruitfly could.)

After generating gene clusters that each contained “all, and only, those genes that descended from a singe gene in their common ancestor,” the authors used a method to infer the evolutionary relationships of the genes in each cluster. They could then compare these gene trees to the known evolutionary relationships of the organisms to determine when each gene duplicated in relation to when the lineages diverged. From this analysis, Dehal and Boore identified over 3,500 gene duplications present in multiple vertebrates, indicating they had occurred at the base of the vertebrate tree, dating back some 450 million years. But did these early duplication events arise from some large-scale duplication event, or were they simply the result of a great number of smaller scale duplications?

To explore this question, the authors analyzed the relative positions of the resulting paralogs in the vertebrate genome with the highest-quality data—the human genome. When considering only this subset of 3,500-plus early vertebrate duplications, they found a global pattern of human genome segments with similar arrangements of paralogous genes and multiple chromosomes with long linear stretches of interdigitated sets of paralogous genes—evidence that the duplications occurred in large segments. Even stronger support for the 2R hypothesis comes from the observation that the colinear arrangement of these genes is predominantly in a 4-fold pattern; this repetitive pattern is seen across almost all the human chromosomes. It's unlikely, the authors argue, that any combination of smaller, independent duplication events could have generated the same pattern.

Now that strong evidence for Ohno's hypothesis exists, researchers can investigate both the mechanism of genome duplication events and their possible effects on vertebrate evolution. It seems likely that a whole genome duplication would provide combinatorial possibilities that could permit a greater leap in evolution than could single gene duplications, even if the single gene duplications affected the complete set of genes. Studies that examine the function of these paralogous genes can explore whether these large-scale genomic events helped drive organismal complexity and diversification within the vertebrate lineage. —*Liza Gross*

